# Supplementary material for: Resolving the Sodiation Process in Hard Carbon Anodes with Nanostructure Specific X‐Ray Imaging
Source: Adv Sci (Weinh). 2025 Jun 20;12(34):e08635. doi: 10.1002/advs.202508635 (PMC12442671; doi:10.1002/advs.202508635)
Supplement: Supplementary file 1 — Supporting Information [file ADVS-12-e08635-s001.docx]

Supporting information

Resolving the sodiation process in hard carbon anodes with nanostructure specific X-ray imaging

Martina Olsson^1^, Antoine Klein^1^, Nataliia Mozhzhukhina^1,2^, Shizhao Xiong^1^, Christian Appel^3^, Mads Carlsen^3^, Leonard Nielsen^1^, Linnea Rensmo^3,4^, Marianne Liebi^1,3,4^, Aleksandar Matic^1^

*1. Department of Physics, Chalmers University of Technology Gothenburg 41296, Sweden.*

*2. SEEL Swedish Electric Transport Laboratory, Säve Flygplatsväg 27, 42373 Säve, Sweden*

*3. Center for Photon Science, Paul Scherrer Institut, 5232 Villigen PSI, Switzerland*

*4. Institute of Materials, Ecole Polytechnique Fédérale de Lausanne (EPFL), 1015 Lausanne, Switzerland*

a)


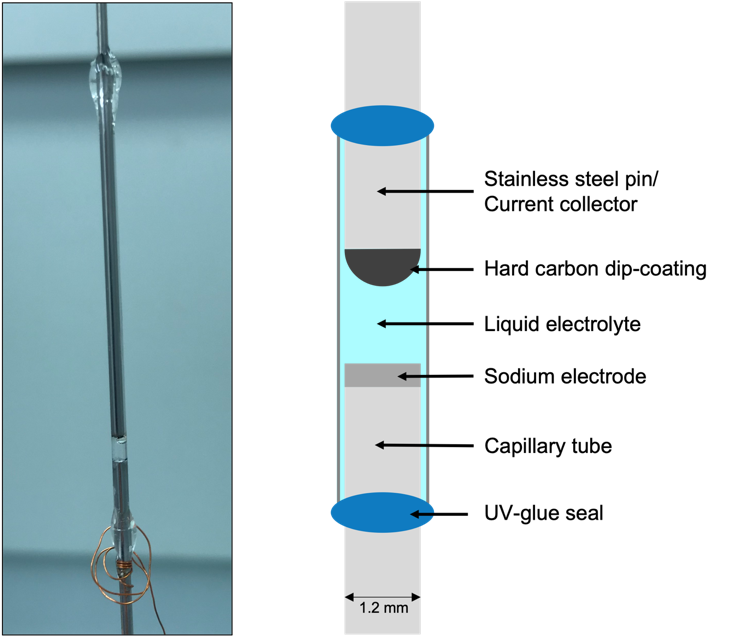

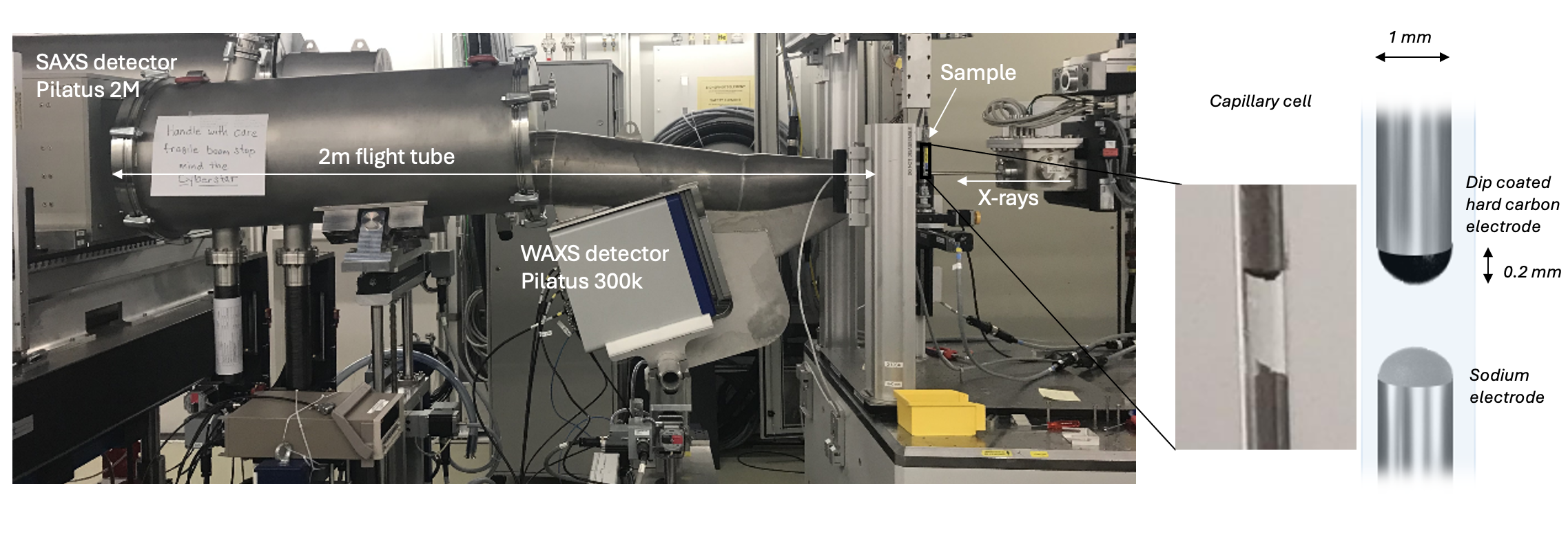


b)

**Figure S1.** **a)** Schematic image of the capillary-based electrochemical cell used in the experiments**. b)** Experimental set up at cSAXS

**Estimation of charge rate for the coated electrodes**

To estimate the charge rate applied to the different electrodes the volume of the electrodes was calculated from the SAXS imaging results to estimate the mass of each electrode. For the *ex situ* samples the number of voxels in each tomogram of the segmented electrode was calculated while for the operando electrode the volume of the electrode was estimated from the area in the scanning SAXS images by assuming symmetry of the spherical cap. The mass was calculated according to equation S1

$mass=\#Voxels*Voxel dimension*Volume fraction HC*density HC$ eq. S1

where the fraction of active hard carbon material is 0.85, the voxel dimension is 15*25*25 μm^3^ and a density of 1.5 g/cm^3^ of the hard carbon. The resulting C-rate applied to the electrode was further calculated according to equation S2

$C-rate=\frac{Applied current}{Specific Capacity*mass}$ eq. S2

where a specific capacity of 300 mAh/g was assumed^1^. The result of the calculations for the charge rate of the three electrodes are summarized in Table S1.

**Table S1.** Calculated mass, applied current and the corresponding theoretical C-rate for the different electrodes

|  | Calculated mass | Applied current | Current density | Theoretical charge rate |
| --- | --- | --- | --- | --- |
| Operando cell | 0.06 mg | 14 μA | 0.23 A/g | 0.8C \| C/1.3 |
| Low degree of sodiation | 0.17 mg | 14 μA | 0.082 A/g | 0.3C \| C/3.5 |
| High degree of sodiation | 0.22 mg | 14 μA | 0.064 A/g | 0.2C \| C/4.5 |

a)

b)


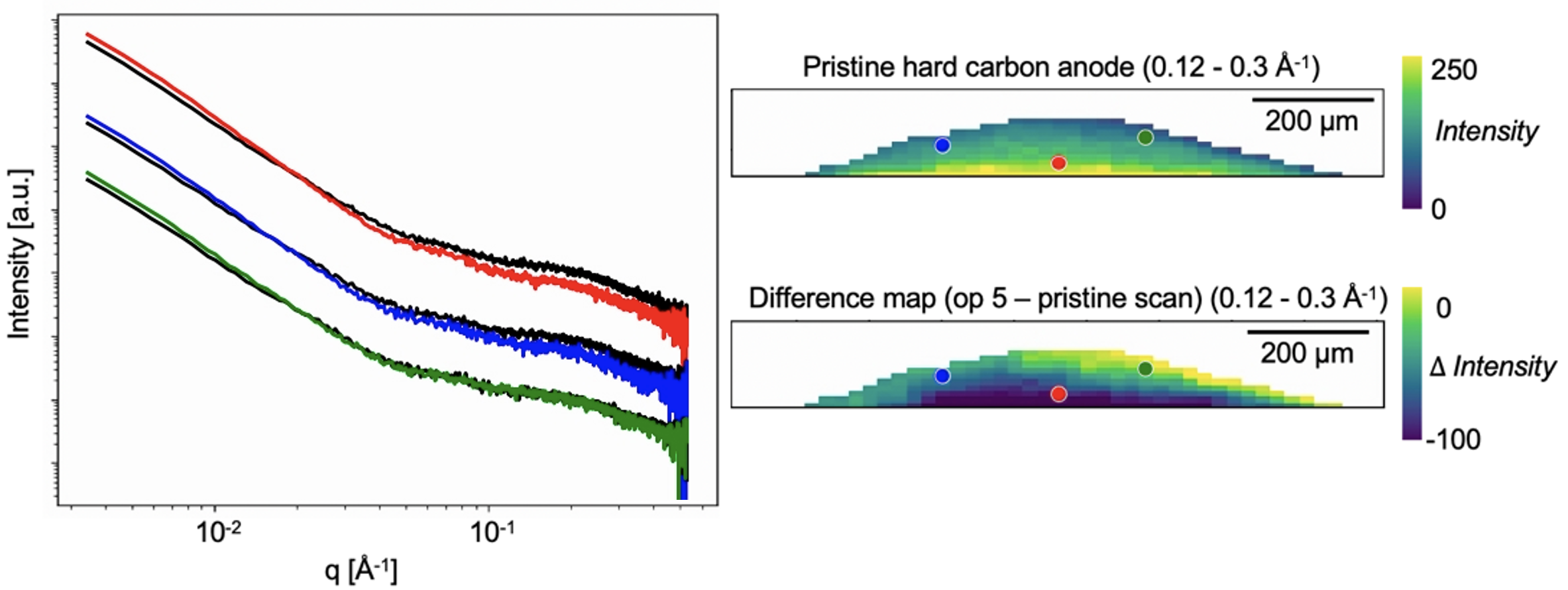


**Figure S2**. Spatially resolved scattering curves at selected points in the pristine and sodiated anode (op 5) **a)** The SAXS curve of the pristine (black) and the sodiated hard carbon (colored) in each point. The coupled curves in each position are offset for clarity. **b)** Scanning SAXS images of the integrated intensity in the microporous (0.12 - 0.3 Å-1) regime of the pristine and sodiated anode (op 5).

**
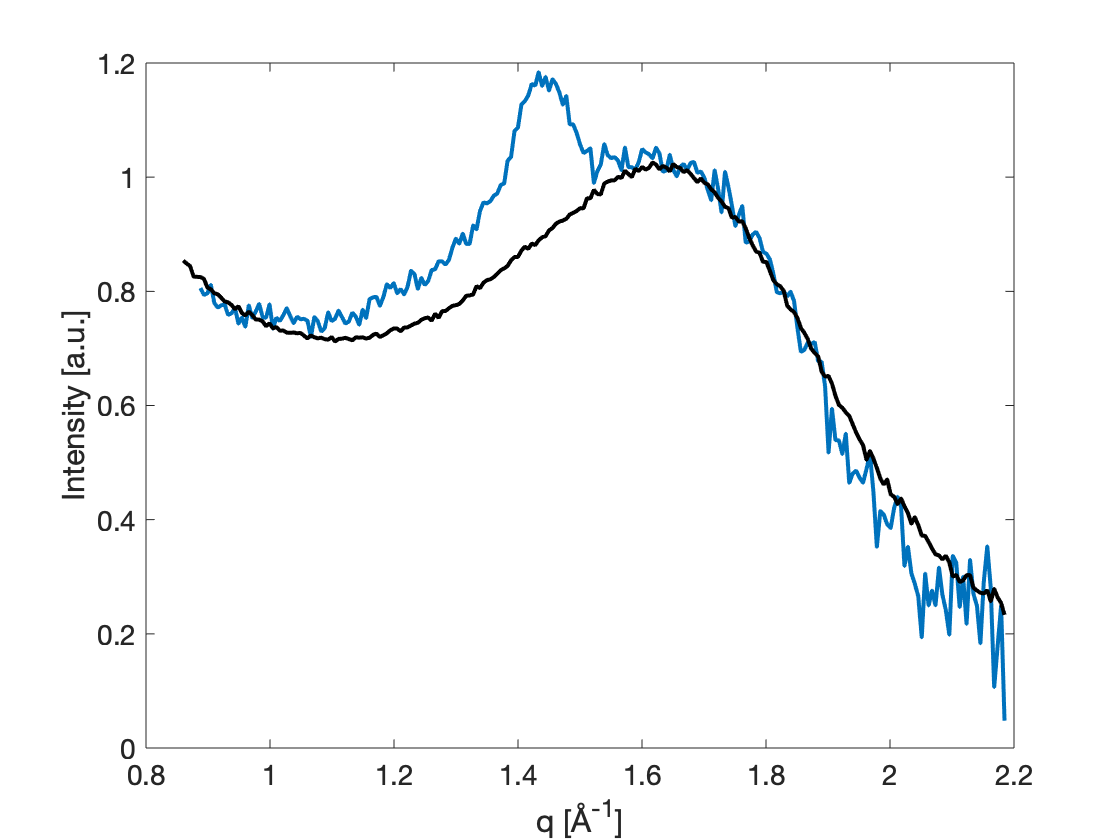
**

**Figure S3.** WAXS signal from an electrode (blue) including hard carbon as well as the inactive binder material and the pristine hard carbon powder (black). The scattering of the electrode is dominated by the broad 002 peak from the hard carbon and a crystalline peak is observed from the inactive binder material at 1.45 Å^-1^.

**WAXS analysis of the hard carbon electrodes**

To analyze the changes in scattering intensity following sodiation the WAXS curve was divided in different q-regions according to the model contribution described in section 2, equation 4. The q-regions were selected to prevent overlap between different contributions, marked in Figure S4a. Apart from analyzing the scattering intensity changes, the WAXS curves were fitted according to the model described in Figure 4. For the first operando scans a linear background, a gaussian curve describing the 002 peak and a gaussian describing the peak from the inactive binder material within the electrode, (fixed peak position q=1.45 Å^-1^) was used to model the curve. In the last operando scan an additional gaussian was added to account for the broad peak from pseudo-metallic sodium appearing. To calculate the average interlayer distance, *d*, Bragg’s law ($d=\frac{2\pi}{q*}$ ) was applied where $q$* is the 002-peak position. The results are shown in Table S2. Due to the high contribution of electrolyte in the cell which overlaps with the 002-peak the background subtraction impose uncertainties for the extraction of the absolute value of the average interlayer distance and the value should be inferred as a parameter showing the relative change rather than the absolute value of interlayer distance.


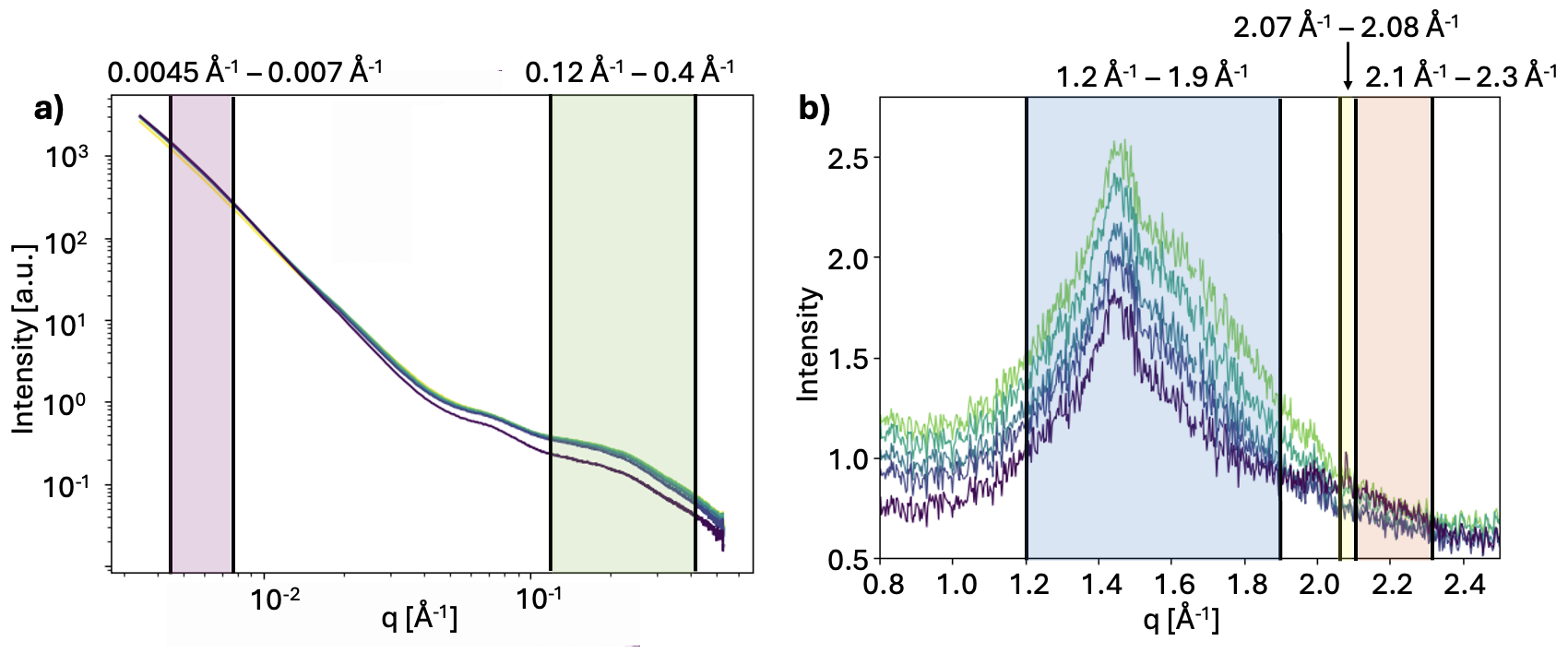

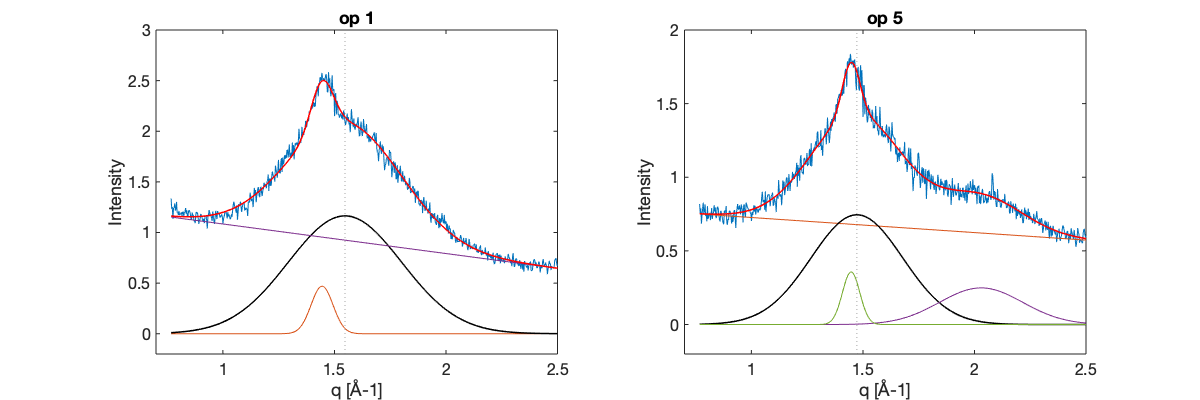

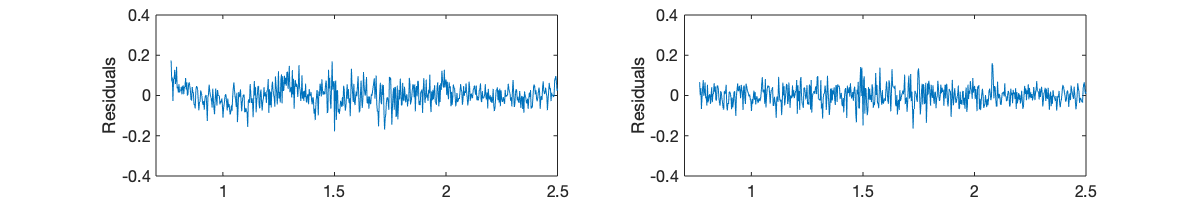


**a)**

**c)**

**b)**

**Figure S4**. **a)** Selected q-regions for intensity analysis in the WAXS region. Fitting results of average WAXS curves from the electrode in the **b)** first operando scan (op 1) and **c)** last operando scan (op 5). The extract 002 peak is shown in black, and the peak position, q*, is marked by a grey dotted line. The fitting was performed with a linear background and a gaussian curve describing the 002 peak as well as a second gaussian describing the peak from the inactive binder material within the electrode, (peak position q=1.45 Å^-1^). In the last operando scan an additional gaussian was added to account for the broad peak of Na-Na correlation from pseudo-metallic sodium confined in the micropores**.**

**Table S2.** Peak position and derived averaged interlayer spacing of the 002-peak in the electrode from the operando WAXS curves. Due to the strong background of electrolyte the values of the average interlayer spacing should be regarded as a relative measurement parameter for comparing the change between scans rather than an absolute measurement.

|  | Peak position 002, q* | Average interlayer spacing, 2$\boldsymbol{\pi}$/q* |
| --- | --- | --- |
| Op 1 | 1.55 Å^-1^ | 4.06 Å |
| Op 2 | 1.53 Å^-1^ | 4.12 Å |
| Op 3 | 1.52 Å^-1^ | 4.14 Å |
| Op 4 | 1.51 Å^-1^ | 4.16 Å |
| Op 5 | 1.47 Å^-1^ | 4.27 Å |


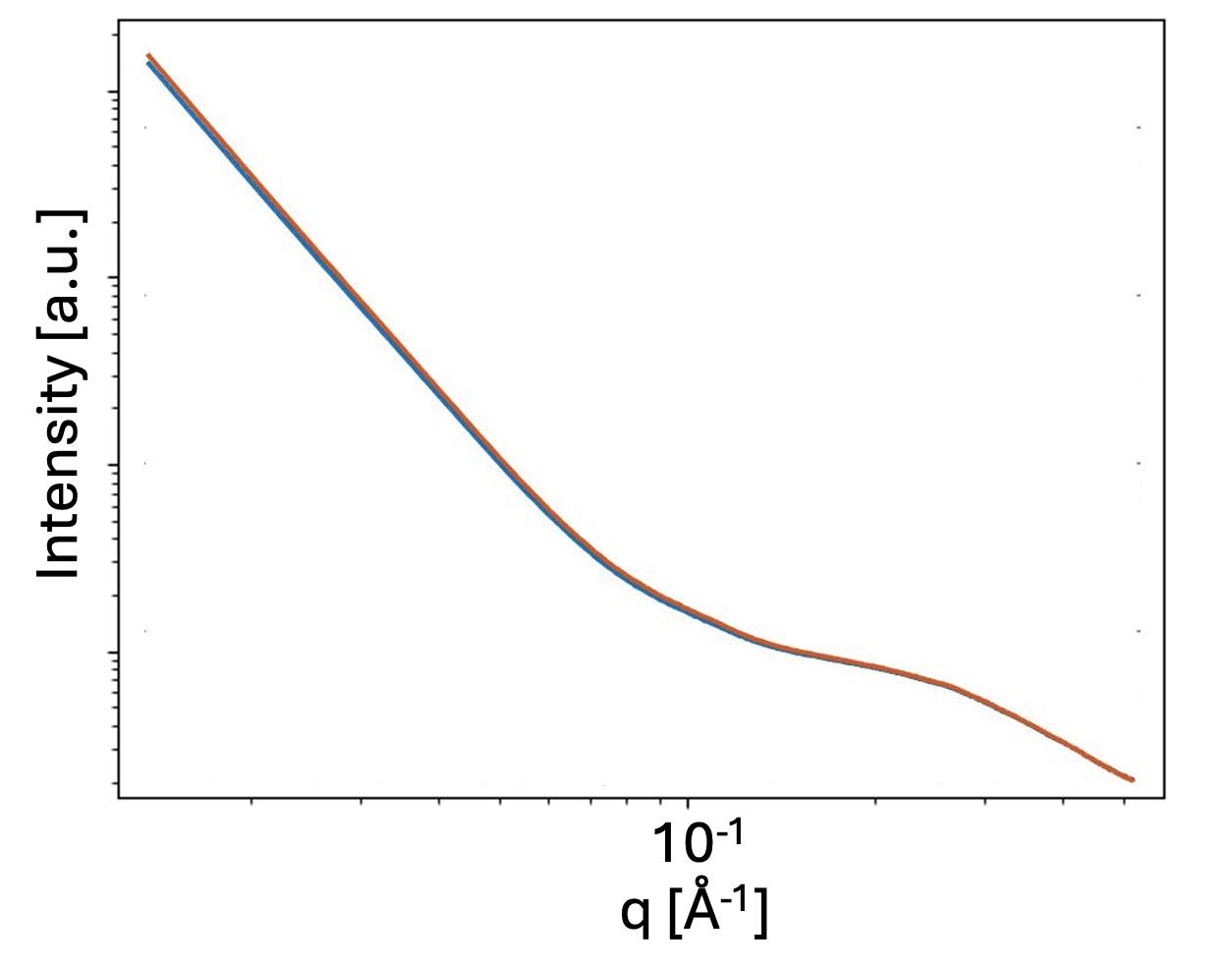


**Figure S5**. Comparison of the average SAXS scattering signal of the first (blue) and last (orange) projection in a tomography measurement of a hard carbon electrode. The curves shows that the radiation dose of the tomogram does not inflict any distinct structural changes and the scattering intensity in the microporous regime remains constant.

(1) Stevens, D.; Dahn, J. High capacity anode materials for rechargeable sodium‐ion batteries. *Journal of the Electrochemical Society* **2000**, *147* (4), 1271.
